# Effects of fasting on thyroid hormone profiles: a systematic review and meta-analysis of TSH, FT3, FT4, and total T3 responses

**DOI:** 10.3389/fendo.2026.1876045

**Published:** 2026-08-06

**Authors:** Süleyman Ulupınar, Kaan Kaya, Ayşe Çamlı, Merve Terzi, Alişan Yavuz, Serhat Özbay

**Affiliations:** 1 Faculty of Sport Sciences, Erzurum Technical University, Erzurum, Türkiye; 2 Faculty of Sport Sciences, Istanbul Yeni Yuzyil University, Istanbul, Türkiye; 3 Faculty of Health Sciences, Department of Nutrition and Dietetics, Erzurum Technical University, Erzurum, Türkiye; 4 Department of Nutrition and Dietetics, Faculty of Health Sciences, Istanbul Yeni Yuzyil University, Istanbul, Türkiye

**Keywords:** fasting, FT3, FT4, thyroid hormones, total T3, TSH

## Abstract

**Background:**

Fasting induces coordinated metabolic and endocrine adaptations, yet its effects on thyroid hormone dynamics remain incompletely characterized. This systematic review and meta-analysis aimed to evaluate the effects of fasting on thyroid-stimulating hormone (TSH), free triiodothyronine (FT3), free thyroxine (FT4), and total triiodothyronine (total T3).

**Methods:**

PubMed, Web of Science, and Scopus were searched for peer-reviewed original research articles published in English between January 2000 and June 2026. Human studies examining fasting interventions lasting ≥ 24 h and reporting pre–post thyroid hormone data in adults were included. Standardized mean changes were calculated as Hedges’ *g* and pooled using random-effects models. Sensitivity analyses were performed using assumed pre–post correlations of *r* = 0.3, 0.5, and 0.7. To preserve statistical independence, one effect estimate per study (corresponding to the longest eligible fasting duration) was retained in the primary analysis. Subgroup analyses compared fasting durations of ≤ 3 days and > 3 days, and random-effects meta-regression examined fasting duration as a continuous moderator.

**Results:**

Fifteen studies were included in the systematic review. Fasting was associated with significant reductions in FT3 (*k* = 7; g = −1.979, 95% CI [−3.054, −0.905], *p* < 0.001), TSH (*k* = 10; *g* = −0.581, 95% CI [−0.882, −0.280], *p* < 0.001), and total T3 (*k* = 4; *g* = −1.337, 95% CI [−1.866, −0.807], *p* < 0.001). No significant change was observed in FT4 (*k* = 7; g = 0.073, 95% CI [−0.237, 0.382], *p* = 0.644). Heterogeneity was considerable for FT3, moderate for TSH and FT4, and low to moderate for total T3. Sensitivity analyses produced comparable estimates. Subgroup analysis indicated a larger FT3 reduction after fasting > 3 days, whereas duration subgroup differences were not significant for TSH, FT4, or total T3. Meta-regression showed no significant linear association between fasting duration and any thyroid outcome.

**Conclusions:**

Fasting produces a biomarker-specific thyroid response characterized by pronounced reductions in FT3 and total T3, a moderate decrease in TSH, and relative stability of FT4. This pattern is more consistent with altered peripheral thyroid hormone metabolism and reduced T4-to-T3 conversion than with a uniform suppression of thyroid hormone production.

## 1 Introduction

Fasting is a potent physiological stimulus that induces coordinated metabolic and hormonal adaptations to maintain energy homeostasis during periods of caloric deprivation (1, 2). In response to reduced nutrient availability, the human body shifts from glucose-dependent metabolism toward increased reliance on lipid oxidation and alternative energy substrates, accompanied by adjustments in endocrine function (3–5). These adaptations are not limited to energy metabolism alone but involve a complex interplay between multiple hormonal systems that regulate substrate utilization, energy expenditure, and metabolic efficiency (6–8). Among these, endocrine responses play a central role in modulating both short-term metabolic flexibility and longer-term energy conservation mechanisms (9–11).

The hypothalamic-pituitary-thyroid axis (HPT) is a key regulator of energy metabolism, coordinating hormonal responses that influence basal metabolic rate, thermogenesis, and substrate utilization (12, 13). Thyroid-stimulating hormone (TSH), released from the anterior pituitary, controls the synthesis and secretion of thyroxine (T4) and triiodothyronine (T3) from the thyroid gland (14, 15). While T4 is produced in greater quantities, triiodothyronine represents the biologically active form that exerts most of the metabolic effects at the cellular level. Circulating free fractions, particularly free triiodothyronine (FT3) and free thyroxine (FT4), together with total triiodothyronine (total T3), are commonly used to assess thyroid function, with peripheral conversion of T4 to T3 serving as a critical regulatory step in metabolic adaptation (12, 13, 16).

The effects of fasting on thyroid hormone dynamics have been investigated across a range of experimental and clinical settings; however, the reported findings remain heterogeneous (7, 11, 17). Several studies have documented a reduction in circulating FT3 following fasting (18, 19), whereas changes in TSH have been inconsistent (20, 21), and FT4 has often been reported as relatively stable (7, 10, 22). These variations may be influenced by differences in fasting duration, study design, participant characteristics, and measurement protocols. Consequently, the direction and magnitude of thyroid hormone responses to fasting are not uniformly established. Because these sources of variation cannot be resolved within individual studies, a quantitative synthesis that models fasting duration explicitly and evaluates heterogeneity formally is required.

Despite the growing body of research, the relationship between fasting duration and thyroid hormone responses has not been systematically quantified. In particular, whether the magnitude of hormonal changes varies according to the length of fasting remains unclear, as individual studies are often limited by small sample sizes, heterogeneous designs, and variability in intervention protocols. To address this gap, the present study aimed to systematically evaluate the effects of fasting on four key thyroid-related biomarkers—TSH, FT3, FT4, and total T3—using a pre–post meta-analytic approach. In addition to estimating pooled effect sizes, the analysis incorporated sensitivity analyses based on different correlation assumptions, a longest-duration rule to ensure statistical independence, and meta-regression to examine the duration–response relationship for each thyroid outcome.

## 2 Methods

### 2.1 Literature search strategy

A systematic literature search was conducted to identify human studies investigating the effects of fasting on thyroid hormone profiles, including thyroid-stimulating hormone (TSH), free triiodothyronine (FT3), free thyroxine (FT4), and total triiodothyronine (total T3). PubMed, Web of Science, and Scopus were searched for peer-reviewed original research articles published in English between January 2000 and June 2026.

The search strategy comprised two principal domains: fasting-related interventions and thyroid-related outcomes. Terms within each domain were combined using the Boolean operator “OR,” and the two domains were combined using “AND.” Search syntax was adapted to the indexing requirements of each database.

Fasting-related search terms included “fasting,” “prolonged fasting,” “water-only fasting,” “water fasting,” “complete fasting,” “short-term starvation,” “starvation,” and “energy restriction.” Thyroid-related terms included “thyroid hormones,” “thyroid-stimulating hormone,” “TSH,” “thyrotropin,” “triiodothyronine,” “T3,” “total triiodothyronine,” “free triiodothyronine,” “FT3,” “thyroxine,” “T4,” “free thyroxine,” and “FT4.” Broad thyroid-related terms were included to identify studies in which eligible hormonal outcomes were reported only in the full text, tables, or **Supplementary Materials**.

Searches were conducted in the title, abstract, and keyword fields, where supported by the respective database. In PubMed, Medical Subject Headings and free-text terms were used, including terms corresponding to “Fasting,” “Thyrotropin,” “Triiodothyronine,” and “Thyroxine.” Filters were applied, where available, to restrict the search to human studies and English-language publications. Studies primarily investigating intermittent fasting, time-restricted eating, Ramadan fasting, fasting-mimicking diets, caloric-restriction diets, or other dietary interventions without a continuous fasting period were excluded during the screening process.

No restrictions concerning participant sex, ethnicity, health status, or fasting duration were applied at the database-search stage. Eligibility requirements relating to participant age, fasting duration, intervention characteristics, study design, and outcome availability were applied during title and abstract screening and full-text assessment.

All identified records were exported to a reference-management system, and duplicates were removed before screening. Two reviewers (SU and AÇ) independently screened titles and abstracts and subsequently assessed potentially eligible full-text articles. Disagreements were resolved through discussion and consensus, with a third reviewer (SÖ) consulted when necessary. The reference lists of included studies and relevant review articles were also screened to identify additional eligible publications.

**Figure 1:**
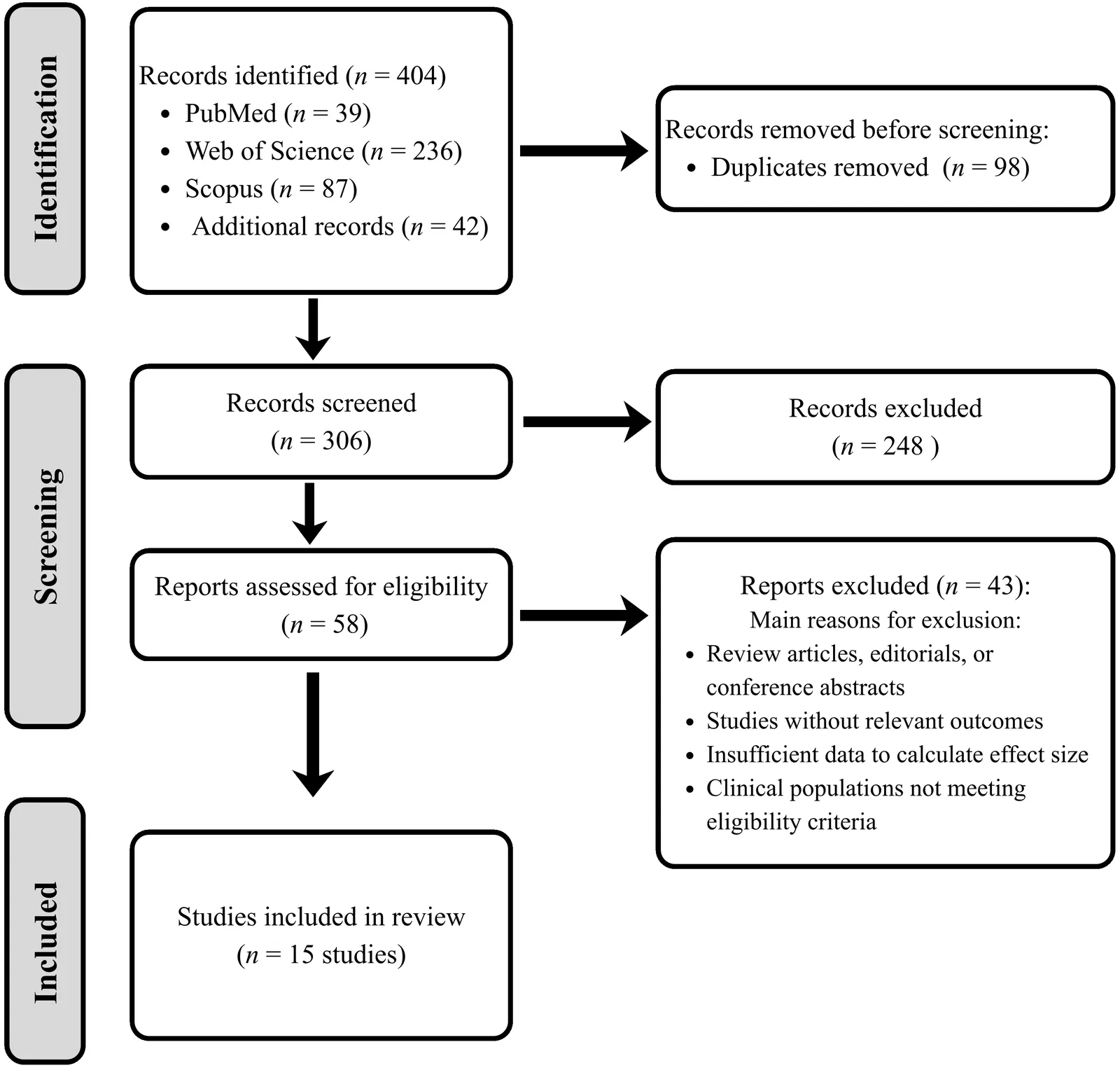
PRISMA 2020 flow diagram of study selection.

The study identification and selection process was conducted in accordance with the PRISMA 2020 guidelines. Complete database-specific search strategies are provided in the **Supplementary Materials**, and the numbers of identified, screened, excluded, and included records are presented in the PRISMA flow diagram (**Figure 1**).

### 2.2 Eligibility criteria

Studies were included if they met all of the following predefined criteria:

#### 2.2.1 Inclusion criteria

Population: Studies involving adult participants (≥ 18 years) were eligible, regardless of sex, ethnicity, or baseline health status. Both healthy individuals and participants with metabolic conditions were included, provided that thyroid hormone outcomes were reported independently of pharmacological or surgical interventions affecting thyroid function.

Intervention: Only studies implementing fasting interventions were included. Eligible protocols required a clearly defined fasting period with no caloric intake. Water-only fasting and prolonged fasting protocols were considered eligible, while non-caloric fluids (e.g., water or electrolytes) were permitted. Interventions based solely on caloric restriction, fasting-mimicking diets, or feeding-based protocols (e.g., intermittent fasting, time-restricted feeding, or Ramadan fasting) were excluded. A minimum fasting duration of 24 hours was required.

Study design: Eligible designs included randomized controlled trials, non-randomized trials, and longitudinal pre–post intervention studies reporting within-subject changes. Both controlled and single-arm studies were accepted, provided that pre- and post-intervention measurements were available.

Outcomes: Studies were required to report at least one of the following thyroid-related biomarkers measured before and after the fasting intervention: TSH, FT3, FT4 or total T3. Studies were included if sufficient information was available to compute standardized effect sizes.

Data availability: Studies were included if they provided adequate quantitative data for effect size calculation, including means and standard deviations or data convertible from standard errors, confidence intervals, medians with interquartile ranges, or graphical representations. When necessary, numerical data were extracted from figures using established digitization methods.

### 2.3 Data extraction and transformation

Two independent reviewers (K.K. and A.Ç.) extracted data from all eligible studies using a standardized data extraction form. Extracted variables included study characteristics (author, year, country), participant characteristics (sample size, age, sex, health status), fasting protocol details (type and duration), and thyroid hormone outcomes (TSH, FT3, FT4 and total T3). Discrepancies were resolved through discussion and consensus.

For each study, mean and standard deviation (*SD*) values for pre- and post-fasting measurements were extracted whenever available. When data were reported in alternative formats, standard transformation procedures were applied to ensure comparability across studies.

When results were reported as mean ± standard error (*SE*), *SD* was calculated using the formula:


$$SD=SE\times \sqrt{n}$$

For studies reporting medians and interquartile ranges (IQR), mean and *SD* values were estimated using established methods Hozo et al. (2005) and refined by Wan et al. (2014) (23, 24).

When numerical values were not directly reported, data were extracted from graphical representations using WebPlotDigitizer (version 4.x) (25, 26). To minimize extraction error, digitization was performed independently by two reviewers and averaged.

Because most studies did not report the correlation between pre- and post-intervention measurements, a correlation coefficient of *r* = 0.5 was assumed for the calculation of within-subject standardized mean differences. Sensitivity analyses were conducted using alternative assumptions (*r* = 0.3 and 0.7) to assess the robustness of the results.

Fasting duration was standardized across studies by converting all reported units (hours or days) into days. For studies reporting duration ranges, the midpoint value was used.

When multiple effect estimates were reported within a single study (e.g., multiple experimental conditions or repeated measurements), effect sizes were calculated separately for each outcome (TSH, FT3, FT4 and total T3) and combined within the study using inverse-variance weighting when necessary to maintain statistical independence in the primary meta-analysis.

### 2.4 Effect size calculation

Effect sizes were calculated as Hedges’ *g*, representing the standardized difference between post-fasting and pre-fasting measurements. Hedges’ *g* was selected to reduce small-sample bias and permit the synthesis of thyroid outcomes reported in different measurement units.

For each study, the pre–post mean difference was standardized using the pooled pre- and post-intervention standard deviation. A small-sample correction was subsequently applied to obtain Hedges’ *g*. Sampling variances incorporated the assumed correlation between pre- and post-fasting measurements. Because this correlation was not reported in most studies, a value of (*r* = 0.5) was used in the primary analysis. Sensitivity analyses were conducted using alternative assumptions of (*r* = 0.3) and (*r* = 0.7).

Effect sizes were oriented so that positive values indicated an increase and negative values indicated a decrease following fasting. This orientation was applied consistently to thyroid-stimulating hormone, free triiodothyronine, free thyroxine, and total triiodothyronine.

Effect sizes were calculated and analyzed separately for each thyroid outcome. Accordingly, a study reporting more than one eligible biomarker could contribute one effect estimate to each relevant outcome-specific meta-analysis.

To preserve statistical independence, only one study-level effect estimate was retained per thyroid outcome in the primary meta-analysis. When a study reported multiple experimental conditions or independent subgroups at the same fasting duration, the corresponding estimates were combined using inverse-variance weighting. For repeated conditions involving the same participants, the sample size was not summed. For independent subgroups, subgroup sample sizes were combined as appropriate.

When multiple fasting-duration measurements were available for the same participants, the estimate corresponding to the longest eligible fasting duration was retained for the primary study-level analysis. Duration-specific estimates were retained separately only for the meta-regression dataset and were analyzed using study-level cluster-robust standard errors.

### 2.5 Statistical analysis

All analyses followed established methods for the quantitative synthesis of pre–post intervention studies. Four thyroid outcomes—thyroid-stimulating hormone, free triiodothyronine, free thyroxine, and total triiodothyronine—were analyzed in separate outcome-specific models. Standardized pre–post effects were expressed as Hedges’ *g*, with negative values indicating a decrease and positive values indicating an increase following fasting.

Pooled effect sizes were estimated using random-effects models to account for within-study sampling error and between-study heterogeneity. The primary analysis used the DerSimonian–Laird estimator for the between-study variance. Restricted maximum likelihood and Paule–Mandel estimates were also calculated to evaluate the robustness of the heterogeneity estimates. Hartung–Knapp-adjusted confidence intervals based on the REML estimator were calculated as supplementary robustness estimates, whereas the primary pooled estimates and confidence intervals were derived from the DerSimonian–Laird model. Ninety-five percent prediction intervals were also calculated to describe the expected range of effects in a future comparable study.

To maintain statistical independence, multiple estimates from the same study were aggregated before the primary meta-analysis. Where required, estimates from multiple conditions or subgroups were combined using inverse-variance weighting to obtain one study-level effect estimate per thyroid outcome. When the same participants were assessed after multiple fasting durations, the longest eligible fasting duration was retained in the primary study-level analysis. A study reporting more than one thyroid outcome could contribute separately to each relevant outcome-specific model.

Between-study heterogeneity was evaluated using Cochran’s *Q* statistic, (*I*²), and (τ²). Approximate (*I*²) values of 25%, 50%, and 75% were interpreted as indicating low, moderate, and high heterogeneity, respectively. The statistical significance of Cochran’s *Q* was also reported.

The primary analyses assumed a pre–post correlation coefficient of (*r* = 0.5). Sensitivity analyses were conducted using alternative correlation assumptions of (*r* = 0.3) and (*r* = 0.7) to evaluate whether uncertainty regarding the within-participant correlation materially affected the pooled estimates.

Exploratory subgroup analyses were conducted according to fasting duration, categorized as 3 days or less and more than 3 days. Random-effects estimates were calculated within each subgroup, and differences between duration categories were evaluated using a between-subgroup *Q* test. Subgroup findings were interpreted cautiously because of the small number of studies in some categories.

The association between fasting duration and effect size was further examined using random-effects meta-regression, with fasting duration in days entered as a continuous moderator. Separate models were fitted for each thyroid outcome using inverse-variance-weighted estimation and study-level cluster-robust standard errors. Regression coefficients, robust standard errors, 95% confidence intervals, t statistics, and corresponding p-values were reported. Meta-regression results, particularly for outcomes with few studies, were considered exploratory.

Potential small-study effects were assessed by visual inspection of funnel plots. Egger’s regression test was performed only when at least 10 independent study-level effect estimates were available for an outcome. Funnel plots and Egger test results were interpreted cautiously because of the limited number of studies and the potential influence of between-study heterogeneity.

All analyses were performed using Python 3 with custom-written scripts using the pandas, NumPy, SciPy, and Matplotlib libraries.

### 2.6 Risk of bias considerations

The methodological quality of the included studies was evaluated using the National Institutes of Health Quality Assessment Tool for Before–After (Pre–Post) Studies With No Control Group. The tool was applied consistently to the pre–post fasting comparison extracted from each study, including studies employing randomized, controlled, or crossover designs.

The assessment covered 12 domains: clarity of the study objective; description of eligibility criteria; representativeness of the study population; enrollment of eligible participants; adequacy of the sample size; clarity and consistency of the fasting intervention; prespecification, validity, and reliability of outcome measures; blinding of outcome assessors; completeness of follow-up; appropriateness of pre–post statistical analyses; use of repeated outcome measurements; and appropriateness of the group-level analysis.

Two reviewers independently evaluated each study. Individual domains were classified as “Yes” when the criterion was adequately met, “No” when it was not met or represented an important methodological concern, “Cannot determine” when the relevant information was insufficiently reported, or “Not applicable” when the criterion was not relevant to the study. Because the outcomes included in this review were objective laboratory measurements, assessor blinding was generally classified as not applicable unless the study design indicated otherwise.

An overall methodological quality rating of “Good,” “Fair,” or “Poor” was assigned based on the domain-level assessments and an overall consideration of study design, sample size, participant flow, intervention reporting, outcome measurement, and potential sources of bias. No numerical cutoff or unweighted domain score was used to determine the overall rating. Randomized or crossover designs were considered supportive of internal validity, whereas small samples, incomplete reporting, absence of a concurrent control, heterogeneous populations, and relevant clinical co-interventions were considered potential limitations. Information not reported in the available study description was not automatically treated as evidence that a criterion had not been met.

Assessors were not blinded to study authorship, journal, or institution. Disagreements between reviewers were resolved through discussion and consensus, with a third reviewer (SÖ) consulted when agreement could not be reached; domain-level judgments were never averaged numerically. Methodological quality ratings were used to characterize the strength and limitations of the available evidence and were not used as criteria for study exclusion. Detailed domain-level judgments and overall quality classifications for all included studies are presented in **Supplementary Table 1**.

## 3 Results

### 3.1 Study selection

The literature search identified 404 records, including 39 from PubMed, 236 from Web of Science, 87 from Scopus, and 42 from additional sources. After removal of 98 duplicate records, 306 unique records remained for title and abstract screening. Of these, 248 records were excluded, and 58 full-text reports were assessed for eligibility. Following full-text evaluation, 43 reports were excluded because they were review articles, editorials, or conference abstracts; did not report relevant thyroid outcomes; provided insufficient data for effect-size calculation; or included clinical populations that did not meet the predefined eligibility criteria. Consequently, 15 studies met the inclusion criteria and were included in the systematic review. The study selection process is presented in the PRISMA 2020 flow diagram (**Figure 1**).

### 3.2 Study characteristics

A total of 15 studies were included in the systematic review, and the principal study characteristics are summarized in **Table 1**. The included evidence comprised randomized crossover studies, prospective interventional and longitudinal studies, single-arm repeated-measures designs, and one randomized pilot trial. Sample sizes ranged from 5 to 58 participants. Most studies enrolled healthy adults, whereas two studies included clinical populations: men with type 2 diabetes mellitus and women with HER2-negative stage II–III breast cancer undergoing chemotherapy. Several studies were restricted to either men or women, while others included both sexes.

**Table 1:** Characteristics of included studies, including study design, participant profiles, fasting protocols, thyroid-related outcomes.

Author (year), country	Study design & population characteristics	Fasting protocol & duration	Relevant thyroid outcomes reported
Basolo et al. (2019), USA (32)	Randomized crossover metabolic study involving 58 healthy, euthyroid adults with normal glucose regulation (46 men, 12 women; age, 36.5 ± 10.1 years; BMI, 26.2 ± 4.0 kg/m²). Participants were weight-stable and free from major medical conditions.	Participants completed a 24-h water-only fast under controlled conditions in a whole-room indirect calorimeter. Blood samples were obtained immediately before and after fasting. Dietary conditions were separated by 3-day washout periods.	FT3 decreased from 2.89 to 2.72 pg/mL (−5.7%; p = 0.001), whereas FT4 increased from 1.23 to 1.33 ng/dL (+8.3%; p < 0.0001). TSH decreased from 3.89 to 2.06 µIU/mL (−43.3%; p < 0.0001).
Cagigas et al. (2025), USA (33)	Single-arm longitudinal study involving 20 adults (55% women; age, 52.2 ± 11.8 years; BMI, 28.8 ± 6.4 kg/m²). The cohort included normal-weight, overweight or hyperglycemic, and obese participants.	Participants underwent medically supervised water-only fasting for 9.8 ± 3.1 days, followed by 5.3 ± 2.4 days of gradual plant-based refeeding. Assessments were performed at baseline, at the end of fasting, and after refeeding.	FT3 decreased from 3.29 ± 0.35 pg/mL at baseline to 2.05 ± 0.39 pg/mL at the end of fasting.
Chan et al. (2003), USA (27)	Randomized, double-blind crossover study involving six healthy, lean men (age, 23.3 ± 1.2 years; BMI, 23.7 ± 0.6 kg/m²).	Participants completed a 72-h water-only fast under controlled conditions.	total T3 decreased from 69.1 ± 9.2 to 51.7 ± 19.2 ng/dL.
Chan et al. (2006), USA (18)	Randomized, double-blind crossover study involving seven healthy young women (age, 22.4 ± 1.2 years) without known immunologic or endocrine disorders.	Participants completed a 72-h water-only fast under controlled conditions.	FT3 decreased from 2.98 ± 0.17 to 1.66 ± 0.11, whereas FT4 changed minimally from 1.22 ± 0.09 to 1.18 ± 0.08.
Gälman et al. (2008), Sweden (20)	Prospective interventional study involving seven healthy adults (three men and four women).	Participants completed a 48-h water-only fast.	TSH decreased from 1.75 ± 0.20 to 0.62 ± 0.04.
Hanssen et al. (2015), The Netherlands (22)	Randomized crossover study involving 16 healthy young adults (eight men and eight women; age, 21.9 ± 3.1 years; BMI, 21.3 ± 1.5 kg/m²; body fat, 20.8 ± 7.8%).	Participants completed a 54-h fast under thermoneutral and mild-cold conditions.	TSH decreased under both thermoneutral conditions (2.0 ± 1.0 to 1.1 ± 0.6) and mild-cold exposure (1.9 ± 1.1 to 1.3 ± 0.7). FT4 changed minimally under thermoneutral conditions (14.5 ± 1.5 to 14.1 ± 2.2) and remained stable during mild-cold exposure (14.6 ± 1.8 to 14.7 ± 2.2).
Jiang et al. (2021), China (19)	Prospective single-arm clinical trial involving 41 healthy, normal-weight adults (17 men and 24 women).	Participants completed a 5-day water-only fast.	FT3 decreased from 4.81 ± 0.86 to 2.32 ± 0.54, whereas FT4 increased from 16.31 ± 2.75 to 17.35 ± 2.71.
Lauc et al. (2026), Croatia (34)	Single-arm longitudinal pilot study involving five healthy White adults aged 40–65 years (four men and one woman). Individuals with acute or chronic disease or ongoing treatment were excluded.	Participants completed a 72-h water-only fast. Blood samples were obtained before fasting, immediately after fasting, and following 11 days of refeeding.	Median TSH decreased from 1.55 to 0.75 mU/L, and median FT3 decreased from 4.53 to 3.05 pmol/L. Median FT4 increased from 13.3 to 14.5 pmol/L, with no significant overall time effect. Values were reported as medians with minimum–maximum ranges; total T3 was not reported.
Mahadevan et al. (2017), India (9)	Prospective study involving 47 adults without known thyroid disorders who completed the analysis (total recruited sample, n = 52).	Participants completed a 24-h fast.	TSH decreased from 2.93 ± 1.62 to 2.46 ± 1.32.
Nuttall et al. (2018), USA (11)	Randomized crossover study involving seven men with type 2 diabetes mellitus.	Participants completed a 72-h fast under controlled conditions.	TSH decreased from 2.1 ± 0.7 to 1.9 ± 0.6, and total T3 decreased from 113 ± 8 to 93 ± 5. In contrast, FT4 increased from 1.00 ± 0.10 to 1.20 ± 0.08.
Pietzner et al. (2024), Germany (21)	Prospective longitudinal interventional study involving 12 apparently healthy adults, predominantly of White European ancestry (seven men and five women), without known disease.	Participants completed a 7-day water-only fast.	TSH remained stable (1.58 ± 0.85 to 1.61 ± 0.79), whereas FT3 decreased from 4.46 ± 1.20 to 2.88 ± 0.64 and FT4 decreased from 15.9 ± 2.3 to 14.6 ± 2.3.
Rubio-Aliaga et al. (2011), Scotland (28)	Single-arm repeated-measures study involving 10 adults (three men and seven women; age range, 25–56 years; BMI range, 18.5–39.7 kg/m²).	Participants completed a 36-h fast under controlled conditions.	TSH decreased from 3.53 ± 3.99 to 1.83 ± 1.67.
Snel et al. (2012), The Netherlands (7)	Randomized controlled crossover study involving 12 healthy, normoglycemic young men (age range, 18–30 years; mean age, 22 years; BMI range, 20–25 kg/m²).	Participants completed a 60-h fast under controlled conditions.	TSH decreased from 2.92 ± 0.54 to 1.26 ± 0.19, and total T3 decreased from 1.82 ± 0.08 to 1.14 ± 0.08. FT4 remained stable (16.53 ± 0.60 to 16.60 ± 0.70).
Yang et al. (2021), China (10)	Prospective within-subject study involving 13 healthy men (age range, 28–55 years; mean age, 39.62 ± 8.13 years).	Participants completed a 10-day fast under controlled conditions.	TSH decreased from 2.8 ± 1.4 to 2.1 ± 1.6. Total T3 decreased from 1.8 ± 0.2 to 0.8 ± 0.2, and FT3 decreased from 5.2 ± 0.5 to 2.8 ± 0.6. FT4 remained stable (18.2 ± 2.1 to 18.0 ± 2.3), as did total T4 (99.7 ± 14.1 to 99.2 ± 15.1).
de Groot et al. (2015), The Netherlands (17)	Randomized pilot study involving 13 women with HER2-negative stage II–III breast cancer receiving (neo)adjuvant TAC chemotherapy. Participants were allocated to short-term fasting or non-fasting conditions.	The fasting group completed a 48-h short-term fast, beginning 24 h before and ending 24 h after chemotherapy initiation, under clinical supervision. Thyroid outcomes were assessed after the first 24 h of fasting, immediately before chemotherapy administration.	In the fasting group, TSH decreased from 1.38 ± 0.45 to 0.61 ± 0.14 mU/L, and FT4 decreased from 15.4 ± 1.59 to 13.9 ± 1.63 pmol/L. Total T3 was measured, but numerical pre–post values were not reported.

The fasting interventions were predominantly water-only or complete fasting protocols conducted under controlled laboratory or clinical conditions. Fasting duration ranged from 24 h to 10 days, with most studies examining short- to intermediate-duration fasting of 24–72 h; fewer studies evaluated prolonged fasting lasting 5–10 days. One study additionally included a structured post-fasting refeeding phase, whereas another implemented fasting around chemotherapy administration.

All studies reported at least one of the four meta-analyzed thyroid outcomes (TSH, FT3, FT4, or total T3). TSH, FT3, and FT4 were the most frequently reported outcomes, whereas total T3 was available in fewer studies; total T4 was reported by some studies but was not meta-analyzed. Outcome data were presented primarily as means with standard deviations or standard errors, although one study reported medians with minimum–maximum ranges. The studies were conducted across North America, Europe, Asia, and the Middle East, reflecting variation in participant characteristics, experimental settings, and fasting duration. Biomarker-specific meta-analyses included only studies that provided sufficient quantitative pre–post data for effect-size estimation.

### 3.3 Risk of bias findings

The methodological quality assessment is presented in **Supplementary Table 1**. Of the 15 included studies, 10 were rated as Good and five as Fair; none were classified as Poor. The studies rated as Fair were Cagigas et al. (33), Lauc et al. (34), Mahadevan et al. (9), Rubio-Aliaga et al. (28), and de Groot et al. (17).

Across the included studies, the research objectives were clearly stated, fasting interventions were adequately described and consistently implemented, thyroid outcomes were assessed using prespecified and objective methods, and appropriate pre–post statistical comparisons were generally reported. Group-level analyses were also considered appropriate in all studies. Blinding of outcome assessors was classified as not applicable because the principal outcomes were laboratory-based thyroid hormone measurements.

The most common methodological limitations concerned incomplete reporting of population representativeness, participant enrollment, and attrition, as well as limited or unreported sample-size justification. Several studies included small samples, particularly Lauc et al. (34) and de Groot et al. (17), which reduced the precision of the estimates. Additional concerns included the absence of concurrent control groups in several single-arm studies, restricted sex-specific samples, heterogeneous participant characteristics, and potential treatment-related confounding in the chemotherapy study. Overall, however, the included evidence demonstrated acceptable methodological quality for evaluating within-participant thyroid hormone responses to fasting.

### 3.4 Free triiodothyronine

**Figure 2:**
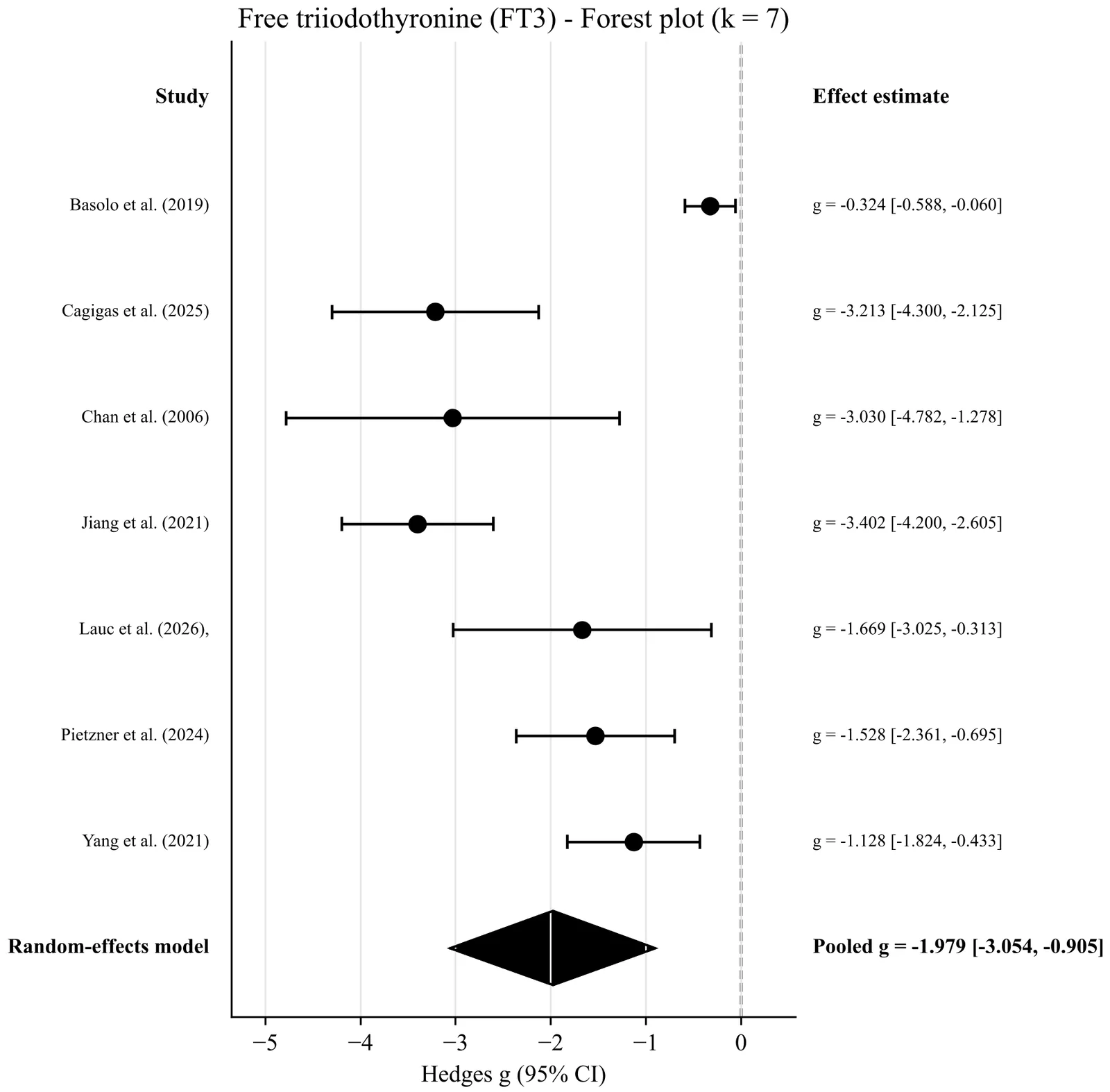
Forest plot of free triiodothyronine (FT3).

A total of seven independent effect sizes were included in the meta-analysis of free triiodothyronine (FT3). Under the primary random-effects model assuming a pre–post correlation of *r* = 0.5, fasting was associated with a significant reduction in FT3 concentrations (Hedges’ *g* = −1.979, *SE* = 0.548, 95% CI [−3.054, −0.905], *p* < 0.001; **Figure 2**).

Between-study heterogeneity was considerable and statistically significant (*Q* = 82.99, *p* < 0.001; *I*² = 92.8%). The estimated between-study variance was τ² = 1.8324 using the DerSimonian–Laird estimator, with corresponding estimates of 1.2062 using Paule–Mandel and 1.3002 using REML. The 95% prediction interval ranged from −5.232 to 1.273, indicating substantial variation in the magnitude of fasting-induced FT3 responses across study settings.

**Figure 3:**
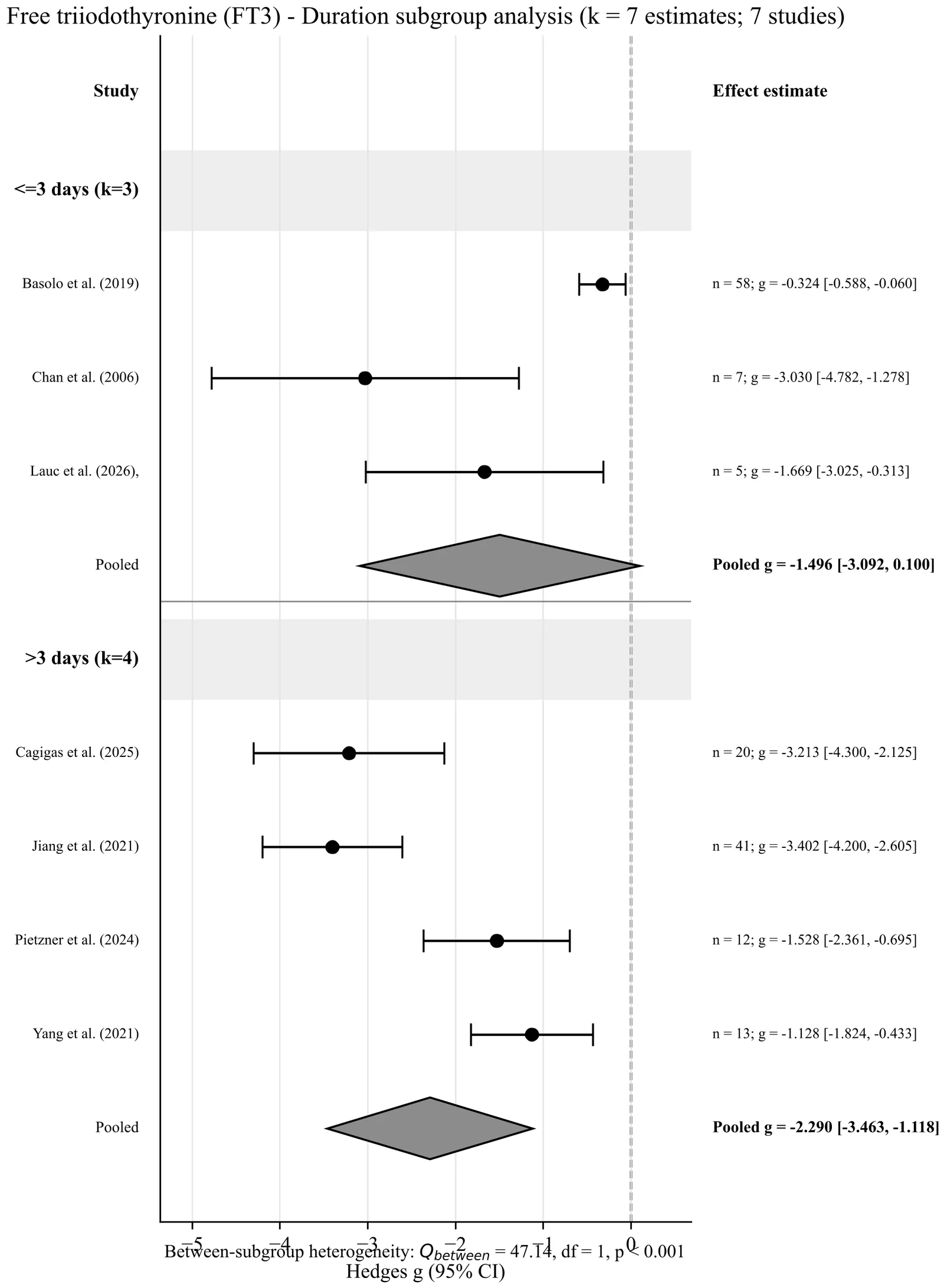
Duration subgroup analysis of free triiodothyronine (FT3).

Sensitivity analyses based on alternative pre–post correlation assumptions produced comparable pooled estimates. The effect size was *g* = −1.981 (95% CI [−3.078, −0.885]) for *r* = 0.3 and *g* = −1.976 (95% CI [−3.019, −0.933]) for *r* = 0.7.Subgroup analyses were conducted according to fasting duration, categorized as ≤ 3 days and > 3 days (**Figure 3**). For fasting interventions lasting ≤ 3 days, the pooled effect was not statistically significant (*k* = 3; *g* = −1.496, 95% CI [−3.092, 0.100]; *I*² = 83.7%). In contrast, fasting interventions lasting > 3 days were associated with a significant reduction in FT3 (*k* = 4; *g* = −2.290, 95% CI [−3.463, −1.118]; *I*² = 87.3%). The difference between duration subgroups was statistically significant (Qbetween = 47.14, *p* < 0.001), suggesting a larger pooled reduction in FT3 following fasting periods exceeding three days.

A random-effects meta-regression with study-level cluster-robust standard errors was performed to examine fasting duration as a continuous moderator. The analysis included seven duration-specific effect estimates nested within seven studies. Fasting duration was not significantly associated with FT3 effect size (β = −0.0882, *SE* = 0.1541, *t* = −0.572, 95% CI [−0.4842, 0.3079], *p* = 0.592). Residual heterogeneity remained after accounting for fasting duration (QE = 3.86, *df* = 5).

Publication bias was evaluated by visual inspection of the funnel plot (**Supplementary Figure 1**). Egger’s regression test was not conducted because fewer than 10 effect sizes were available, limiting the reliability of formal tests for small-study effects.

### 3.5 Free thyroxine

**Figure 4:**
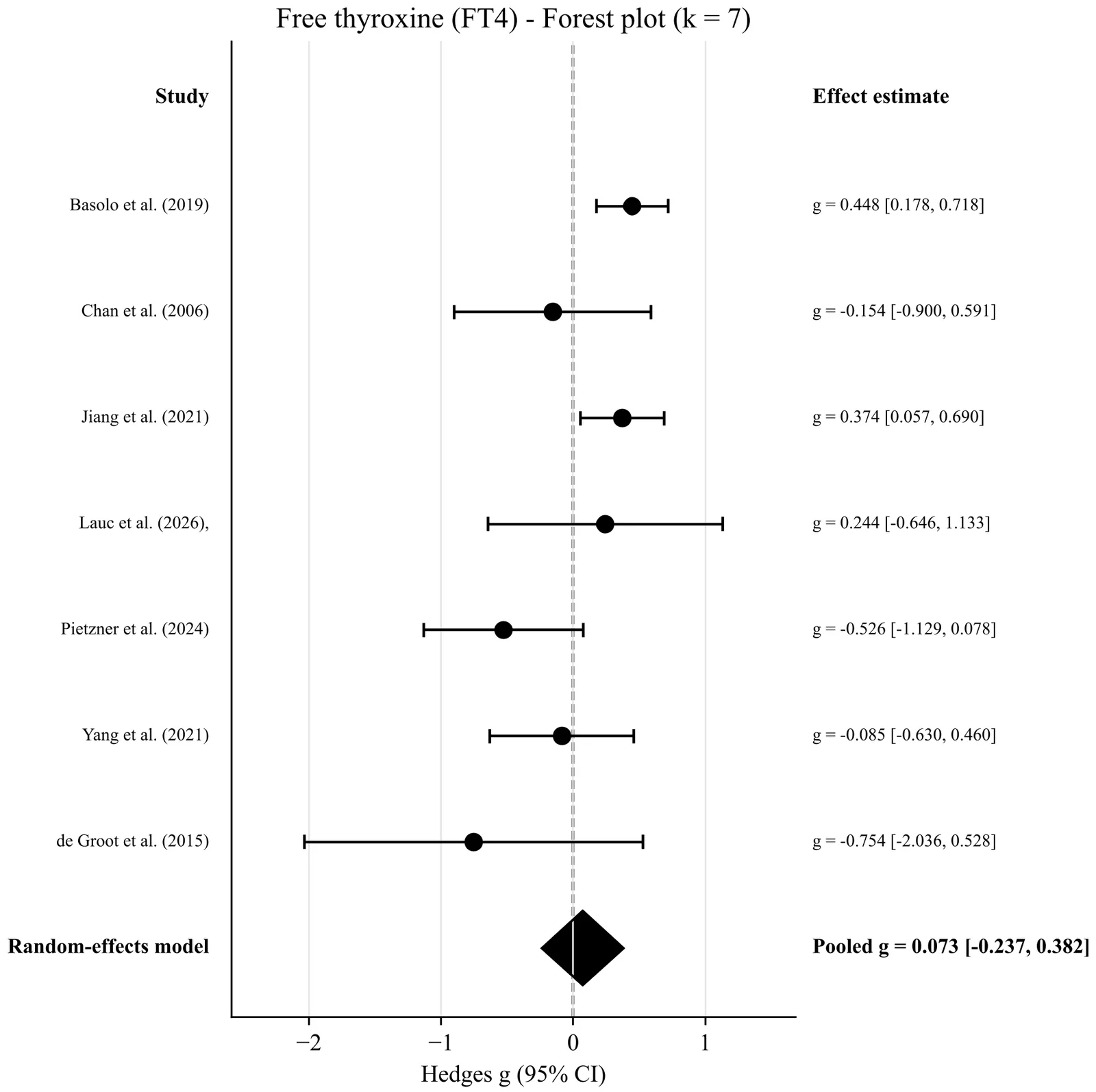
Forest plot of free thyroxine (FT4).

A total of seven independent effect sizes were included in the meta-analysis of free thyroxine (FT4). Under the primary random-effects model assuming a pre–post correlation of *r* = 0.5, fasting was not associated with a statistically significant change in FT4 concentrations (Hedges’ *g* = 0.073, *SE* = 0.158, 95% CI [−0.237, 0.382], *p* = 0.644; **Figure 4**).

Between-study heterogeneity was moderate and statistically significant (*Q* = 13.89, *p* = 0.031; *I*² = 56.8%). The estimated between-study variance was τ² = 0.0850 using the DerSimonian–Laird estimator, with corresponding estimates of 0.0836 using Paule–Mandel and 0.0983 using REML. The 95% prediction interval ranged from −0.830 to 0.975, indicating that the direction and magnitude of FT4 responses varied across studies.

Sensitivity analyses based on alternative pre–post correlation assumptions yielded similar results. The pooled effect was *g* = 0.110 (95% CI [−0.197, 0.417]) for *r* = 0.3 and *g* = 0.037 (95% CI [−0.267, 0.342]) for *r* = 0.7.

**Figure 5:**
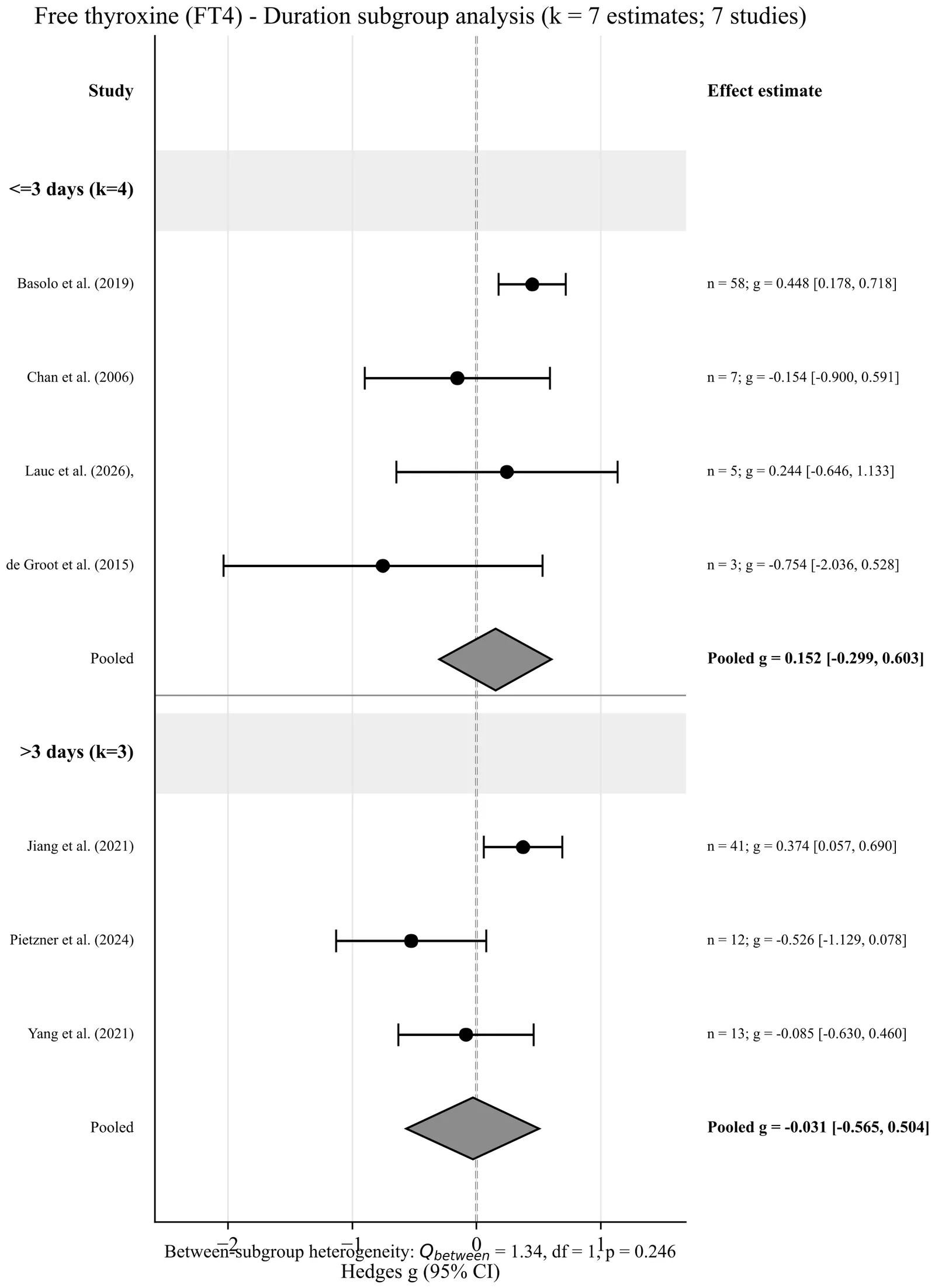
Duration subgroup analysis of free thyroxine (FT4).

Subgroup analyses were conducted according to fasting duration, categorized as ≤ 3 days and > 3 days (**Figure 5**). For interventions lasting ≤ 3 days, the pooled effect was not statistically significant (*k* = 4; *g* = 0.152, 95% CI [−0.299, 0.603]; *I*² = 41.6%). Similarly, fasting interventions lasting > 3 days were not associated with a significant change in FT4 (*k* = 3; *g* = −0.031, 95% CI [−0.565, 0.504]; *I*² = 73.0%). The difference between duration subgroups was not statistically significant (Qbetween = 1.34, *p* = 0.246).

A random-effects meta-regression with study-level cluster-robust standard errors was performed to assess fasting duration as a continuous moderator. The analysis included seven duration-specific effect estimates nested within seven studies. Fasting duration was not significantly associated with FT4 effect size (β = −0.0548, *SE* = 0.0363, *t* = −1.507, 95% CI [−0.1482, 0.0387], *p* = 0.192). Residual heterogeneity remained after accounting for fasting duration (QE = 4.86, *df* = 5).

Publication bias was assessed by visual inspection of the funnel plot (**Supplementary Figure 2**). Egger’s regression test was not performed because fewer than 10 effect sizes were available, limiting the validity of formal small-study-effect testing.

### 3.6 Thyroid-stimulating hormone

**Figure 6:**
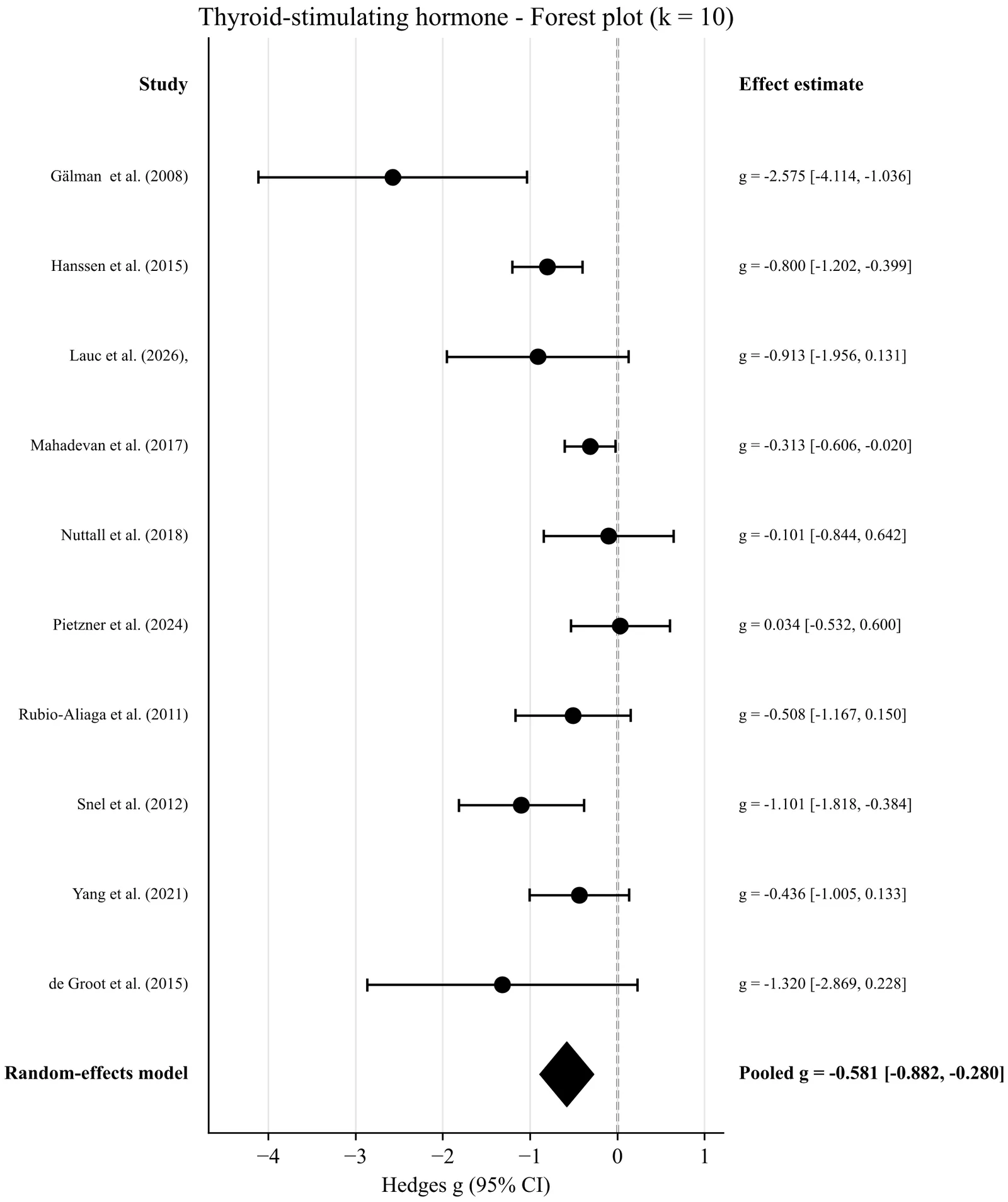
Forest plot of thyroid-stimulating hormone (TSH).

A total of 10 independent effect sizes were included in the meta-analysis of thyroid-stimulating hormone (TSH). Under the primary random-effects model assuming a pre–post correlation of *r* = 0.5, fasting was associated with a statistically significant reduction in TSH concentrations (Hedges’ *g* = −0.581, *SE* = 0.154, 95% CI [−0.882, −0.280], *p* < 0.001; **Figure 6**).

Between-study heterogeneity was moderate and statistically significant (*Q* = 19.66, *p* = 0.020; *I*² = 54.2%). The estimated between-study variance was τ² = 0.1093 using the DerSimonian–Laird estimator, with corresponding estimates of 0.1961 using Paule–Mandel and 0.0870 using REML. The 95% prediction interval ranged from −1.348 to 0.186, indicating variability in the magnitude of TSH responses across studies.

Sensitivity analyses based on alternative pre–post correlation assumptions yielded highly consistent results. The pooled effect was *g* = −0.582 (95% CI [−0.893, −0.271]) for *r* = 0.3 and *g* = −0.574 (95% CI [−0.858, −0.290]) for *r* = 0.7, indicating that the overall findings were robust to different assumptions regarding within-subject correlation.

**Figure 7:**
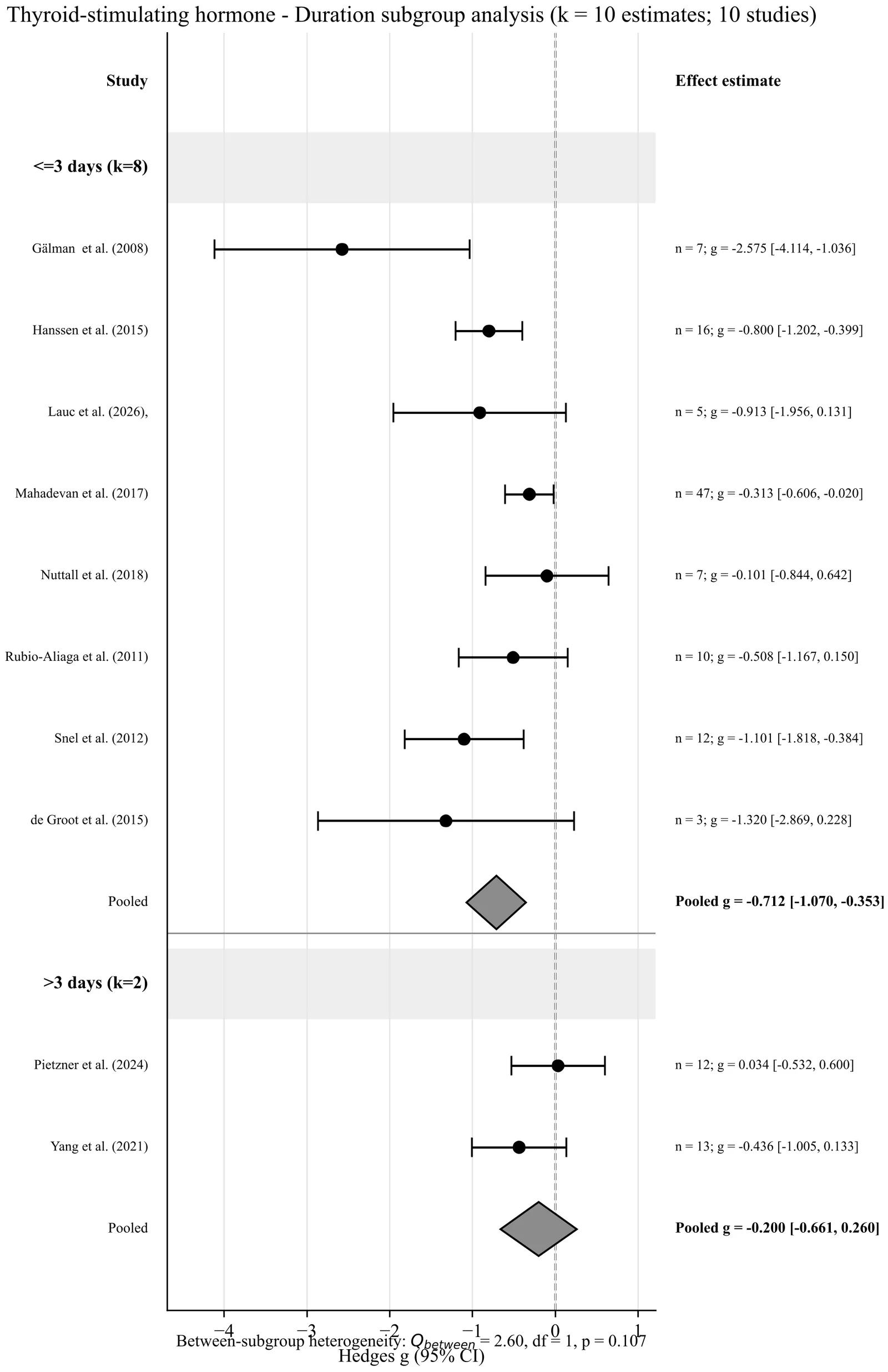
Duration subgroup analysis of thyroid-stimulating hormone (TSH).

Subgroup analyses were conducted according to fasting duration, categorized as ≤ 3 days and > 3 days (**Figure 7**). Fasting interventions lasting ≤ 3 days were associated with a significant reduction in TSH (*k* = 8; *g* = −0.712, 95% CI [−1.070, −0.353]; *I*² = 55.5%). In contrast, fasting interventions lasting > 3 days were not associated with a significant change in TSH (*k* = 2; *g* = −0.200, 95% CI [−0.661, 0.260]; *I*² = 24.1%). The difference between duration subgroups was not statistically significant (Qbetween= 2.60, *p* = 0.107).

A random-effects meta-regression with study-level cluster-robust standard errors was performed to evaluate fasting duration as a continuous moderator. The analysis included 10 duration-specific effect estimates nested within 10 studies. Fasting duration was not significantly associated with TSH effect size (β = 0.0524, *SE* = 0.0421, *t* = 1.246, 95% CI [−0.0446, 0.1494], *p* = 0.248). Residual heterogeneity remained after accounting for fasting duration (QE = 10.48, *df* = 8).

Publication bias was assessed by visual inspection of the funnel plot (**Supplementary Figure 3**) and Egger’s regression test. Egger’s test was not statistically significant (intercept = −1.641, *SE* = 0.935, *t* = −1.76, *p* = 0.117), providing no evidence of small-study effects.

### 3.7 Total triiodothyronine

**Figure 8:**
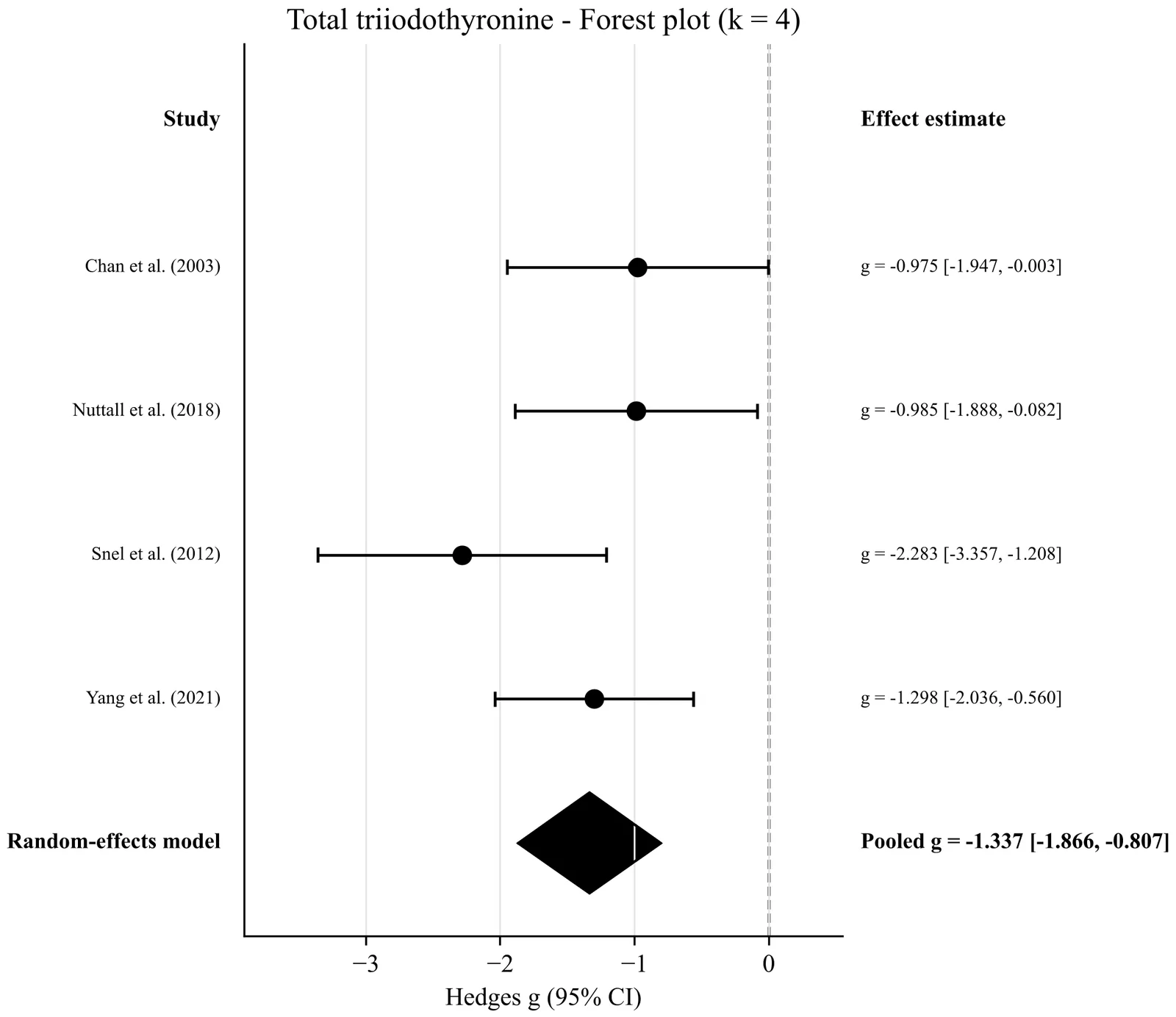
Forest plot of total triiodothyronine (total T3).

A total of four independent effect sizes were included in the meta-analysis of total triiodothyronine (total T3). Under the primary random-effects model assuming a pre–post correlation of r = 0.5, fasting was associated with a statistically significant reduction in total T3 concentrations (Hedges’ *g* = −1.337, *SE* = 0.270, 95% CI [−1.866, −0.807], *p<* 0.001; **Figure 8**).

Between-study heterogeneity was low to moderate and not statistically significant (*Q* = 4.10, *p* = 0.251; *I*² = 26.8%). The estimated between-study variance was τ² = 0.0786 using the DerSimonian–Laird estimator, with corresponding estimates of 0.0945 using Paule–Mandel and 0.0238 using REML. The 95% prediction interval ranged from −2.676 to 0.002, indicating that the direction of effect was predominantly negative, although the upper bound approached the null.

Sensitivity analyses based on alternative pre–post correlation assumptions produced comparable pooled estimates. The pooled effect was *g* = −1.352 (95% CI [−1.895, −0.809]) for *r* = 0.3 and *g* = −1.319 (95% CI [−1.827, −0.810]) for *r* = 0.7.

**Figure 9:**
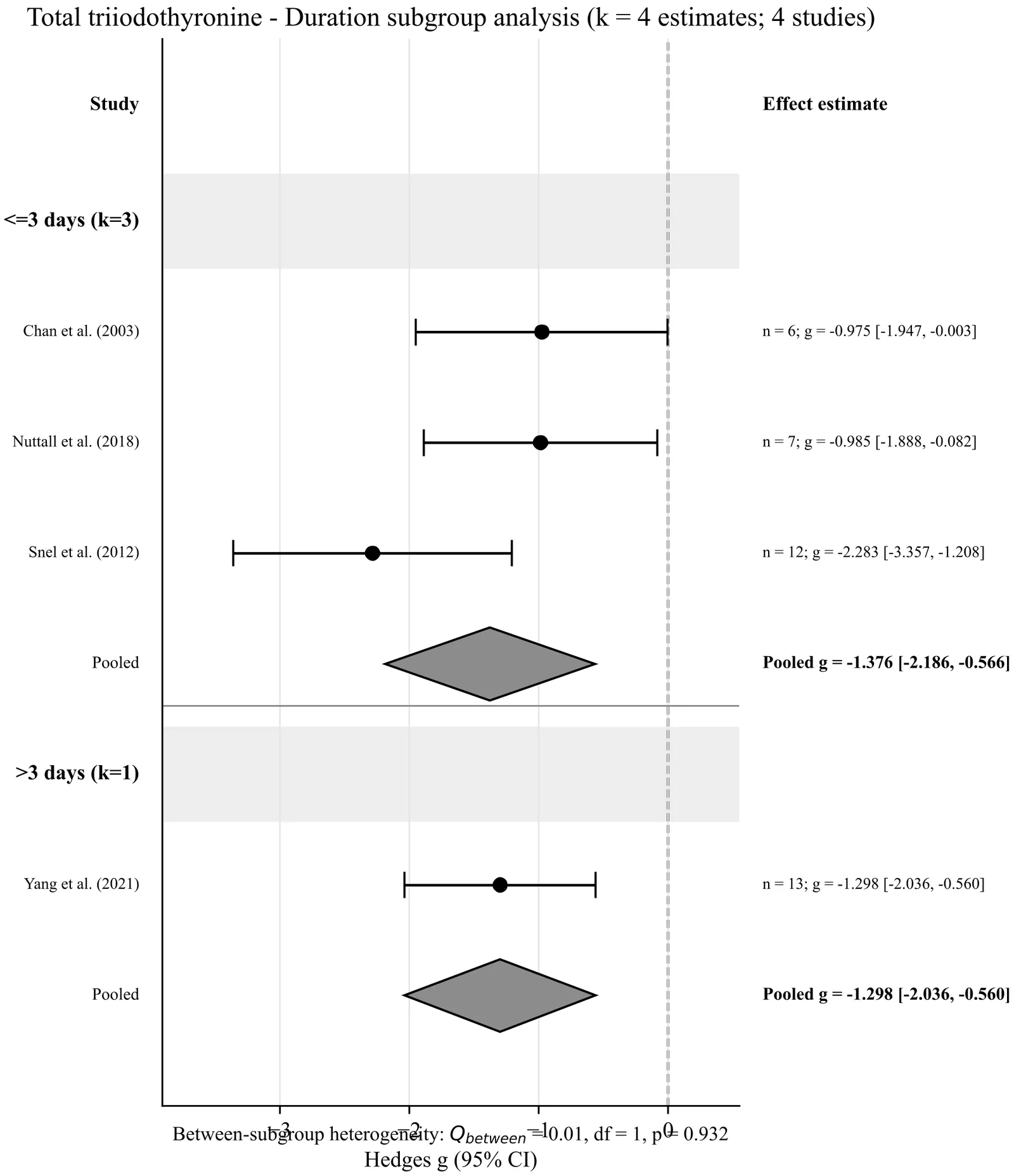
Duration subgroup analysis of total triiodothyronine (total T3).

Subgroup analyses were conducted according to fasting duration, categorized as ≤ 3 days and > 3 days (**Figure 9**). Fasting interventions lasting ≤ 3 days were associated with a significant reduction in total T3 (*k* = 3; *g* = −1.376, 95% CI [−2.186, −0.566]; *I*² = 51.1%). The single study examining fasting lasting > 3 days also showed a significant reduction (*k* = 1; *g* = −1.298, 95% CI [−2.036, −0.560]). The between-subgroup difference was not statistically significant (Qbetween = 0.01, *p* = 0.932). Because the > 3-day subgroup contained only one study, this comparison should be interpreted cautiously.

A random-effects meta-regression with study-level cluster-robust standard errors was performed to evaluate fasting duration as a continuous moderator. The analysis included four duration-specific effect estimates nested within four studies. Fasting duration was not significantly associated with total T3 effect size (β = 0.0159, *SE* = 0.0698, *t* = 0.228, 95% CI [−0.2845, 0.3163], *p* = 0.841). Residual heterogeneity remained after accounting for fasting duration (QE = 3.15, *df* = 2).

Publication bias was assessed by visual inspection of the funnel plot (**Supplementary Figure 4**). Egger’s regression test was not performed because fewer than 10 effect sizes were available, limiting the reliability of formal tests for small-study effects.

## 4 Discussion

The present meta-analysis systematically evaluated the effects of fasting on thyroid hormone profiles and identified a consistent pattern characterized by marked reductions in FT3 and total T3, a moderate decrease in TSH, and no significant change in FT4. These findings were robust across multiple sensitivity analyses, including different pre-post correlation assumptions, and were not materially influenced by publication bias. Collectively, the results suggest that fasting is associated with a selective alteration in thyroid hormone dynamics, with the most pronounced changes observed in the triiodothyronine measures (FT3 and total T3), while FT4 remains relatively stable. This overall pattern is consistent with findings from individual studies, which report reductions in triiodothyronine and variable changes in TSH under controlled fasting conditions.

The pronounced reduction in FT3 observed in the present meta-analysis aligns with findings from several included studies reporting decreases in triiodothyronine following fasting interventions. For example, reductions in FT3 or total T3 were documented in controlled experimental settings such as Chan et al. (2003, 2006) (18, 27), Jiang et al. (2021) (19), Snel et al. (2012) (7), and Yang et al. (2021) (10), as well as in longer fasting protocols reported by Pietzner et al. (2024) (21). This consistent direction of change may reflect alterations in peripheral thyroid hormone metabolism rather than direct changes in thyroid gland output (12, 13, 15). The conversion of T4 to the biologically active T3 is known to be sensitive to energy availability, and reductions in circulating FT3 have been consistently observed under conditions of caloric deprivation (15, 16). Although the overall effect indicates a clear decrease, the magnitude of change varied across studies, which is reflected in the substantial between-study heterogeneity observed in the meta-analysis.

The observed reduction in TSH provides additional support for a coordinated response within the hypothalamic–pituitary–thyroid axis during fasting. Several of the included studies reported decreases in TSH following fasting interventions, including Gälman et al. (2008) (20), Hanssen et al. (2015) (22), Snel et al. (2012) (7), Yang et al. (2021) (10), and Rubio-Aliaga et al. (2011) (28), whereas others noted smaller or negligible changes under similar conditions. This pattern suggests that central regulation of thyroid function may be modulated alongside peripheral hormone metabolism during periods of energy restriction (12, 14). Compared with FT3, the magnitude of change in TSH was more modest, and variability across studies was lower, which is consistent with the heterogeneity estimates observed in the meta-analysis. Taken together, these findings are compatible with the involvement of both central and peripheral components of thyroid regulation, although the extent of change appears to differ between biomarkers.

In contrast to FT3 and TSH, FT4 levels did not show a statistically significant change in the present meta-analysis, a finding also reflected in several of the included studies. For instance, FT4 remained largely stable in controlled fasting protocols reported by Chan et al. (2006) (18), Hanssen et al. (2015) (22), and Snel et al. (2012) (7), while only minor or inconsistent changes were observed in other studies such as Jiang et al. (2021) (19) and Yang et al. (2021) (10). This relative stability of FT4 is consistent with the possibility that fasting-related adaptations may occur primarily at the level of peripheral hormone metabolism rather than through marked alterations in circulating thyroxine levels (13, 16). The consistency of this finding across studies, together with the low-to-moderate heterogeneity observed in the meta-analysis, supports the view that FT4 is less responsive to short- and moderate-term fasting than FT3.

A parallel and statistically significant reduction was observed for total T3 (*g* = −1.337, 95% CI [−1.866, −0.807]), mirroring the direction of the FT3 findings. Importantly, this pooled estimate was accompanied by low-to-moderate, non-significant heterogeneity (*I*² = 26.8%)—the lowest among the outcomes showing a significant change—indicating a more consistent response across studies than that observed for FT3. Reductions in total T3 were documented across the contributing studies at short-to-intermediate durations (Chan et al., 2003 (27); Snel et al., 2012 (7); Nuttall et al., 2018 (11)) and during prolonged fasting (Yang et al., 2021 (10)). The concordant decline in both the free and total fractions of triiodothyronine, together with the relative stability of FT4, is consistent with a fasting-induced downregulation of peripheral T4-to-T3 conversion rather than a reduction in glandular hormone output (13, 16). This configuration—reduced circulating active hormone (T3) with preserved thyroxine—resembles the low-T3 state commonly reported under conditions of restricted energy availability. Nevertheless, because only four studies contributed to this outcome and the upper bound of the prediction interval approached the null (−2.676 to 0.002), the total T3 estimate should be interpreted with appropriate caution.

Across all four biomarkers, the findings suggest a consistent but biomarker-specific response of the thyroid axis to fasting: FT3 and total T3 show the most pronounced decreases, TSH a more moderate reduction, and FT4 remains largely unchanged. Meta-regression analyses did not identify a significant association between fasting duration and changes in any of the examined biomarkers, indicating that a linear relationship between fasting duration and hormonal responses was not observed within the studied range. This overall pattern is consistent with previous findings, which report reductions in triiodothyronine levels, relatively stable thyroxine concentrations, and variable changes in TSH across fasting studies (11–13). However, prior evidence has largely been derived from small and heterogeneous studies, limiting direct comparability. By integrating these findings, the present meta-analysis provides a quantitative synthesis, in which reductions in the triiodothyronine measures (FT3 and total T3) represent the most pronounced hormonal responses—most consistently so for total T3—whereas changes in TSH and FT4 appear more limited.

The present synthesis advances the existing literature in four respects. First, by restricting eligibility to complete, zero-calorie fasting protocols and excluding intermittent fasting, time-restricted eating, Ramadan fasting, and fasting-mimicking diets, it isolates the thyroid response to continuous energy withdrawal from the confounding influence of periodic refeeding. Second, it replaces the qualitative consensus that “T3 falls while T4 is preserved” with a quantitative, biomarker-specific hierarchy of effect magnitudes. Third, the concurrent analysis of both free and total triiodothyronine demonstrates that the response is not an artefact of a single measurement fraction, thereby strengthening the inference of reduced peripheral T4-to-T3 conversion. Fourth, the absence of a linear duration–response relationship across all four outcomes, formally tested by meta-regression, challenges the implicit assumption that longer fasting produces proportionally greater thyroidal suppression.

The present study has several methodological strengths that support the robustness and interpretability of the findings. First, the use of a pre–post meta-analytic design enabled direct assessment of within-subject changes in thyroid hormone levels, thereby reducing the influence of inter-individual variability (29, 30). Second, multiple sensitivity analyses were conducted, including different assumptions for pre-post correlation coefficients, supporting the stability of the findings across analytical conditions. In addition, the application of alternative estimators for between-study variance (DerSimonian-Laird, Paule-Mandel, and REML) and the use of Hartung-Knapp adjustments provided more conservative effect size estimates (29, 31). The inclusion of four thyroid-related biomarkers (TSH, FT3, FT4, and total T3) within a unified analytical framework enabled a comprehensive evaluation of fasting-induced hormonal responses (12, 14). Collectively, these methodological features contribute to the internal consistency and overall reliability of the present meta-analysis.

The included populations differed in sex, age, and baseline health status, and two of the fifteen studies enrolled clinical populations (men with type 2 diabetes mellitus and women receiving chemotherapy for breast cancer). This variation was retained deliberately, because the thyroidal response to energy withdrawal is a conserved adaptive mechanism operating through reduced peripheral deiodination (13, 16), and the direction of change in these studies was consistent with that observed in healthy cohorts. Nevertheless, the magnitude of the response may differ across metabolic and clinical states, and the limited number of studies per outcome precluded formal subgroup analysis by health status. This clinical heterogeneity was accommodated analytically through random-effects modelling and prediction intervals, and remains a limitation of the present evidence base.

Several limitations should be considered when interpreting the findings of this meta-analysis. First, the included studies generally involved small sample sizes (range, 5–58 participants), which may limit the precision of individual effect estimates. Second, variability in study designs, participant characteristics, and fasting protocols likely contributed to between-study heterogeneity, particularly evident in the FT3 analyses. Third, although all included interventions involved continuous fasting without caloric intake, protocols varied with respect to permitted non-caloric fluids, clinical context, and experimental conditions. In addition, the correlation between pre- and post-intervention measurements was not reported in most studies, necessitating the use of assumed values; however, sensitivity analyses using alternative correlations (r = 0.3, 0.5, and 0.7) indicated that this did not materially affect the results. Finally, the limited number of studies for certain outcomes may reduce the statistical power of publication bias assessments and meta-regression analyses. Finally, the present findings apply to the range of fasting durations examined (24 h to 10 days) and should not be extrapolated beyond this window.

## 5 Conclusions

This systematic review and meta-analysis demonstrate that fasting induces a differential response within the thyroid axis, characterized by significant reductions in FT3 and total T3, a moderate decrease in TSH, and no significant change in FT4. These findings indicate that fasting is associated with alterations primarily in peripheral thyroid hormone metabolism rather than thyroid gland output. The consistency of these results across multiple sensitivity analyses supports the robustness of the observed effects, despite variability in study designs and fasting protocols. In addition, the absence of a significant relationship between fasting duration and hormonal changes suggests that these adaptations are not linearly dependent on fasting duration within the examined range. Overall, the present study provides a quantitative synthesis of thyroid hormone responses to fasting, contributing to a clearer understanding of endocrine adaptations during energy deprivation.

## Data Availability

The original contributions presented in the study are included in the article/**Supplementary Material**, further inquiries can be directed to the corresponding author/s.

## Supplementary material

Supplementary Figure 1 Funnel plot for free triiodothyronine (FT3).

Supplementary Figure 2 Funnel plot for free thyroxine (FT4).

Supplementary Figure 3 Funnel plot for thyroid-stimulating hormone (TSH).

Supplementary Figure 4 Funnel plot for total triiodothyronine (total T3).

Supplementary Table 1 NIH quality assessment tool for before-after (pre-post) studies with no control group.
